# lncRNA ZFAS1 promotes intervertebral disc degeneration by upregulating AAK1

**DOI:** 10.1515/med-2022-0530

**Published:** 2022-12-09

**Authors:** Zheng Wang, Bin Liu, Xiangyu Ma, Yu Wang, Wenfeng Han, Liangbi Xiang

**Affiliations:** Department of Orthopedics, General Hospital of Northern Theater Command, Shenyang 110016, Liaoning, China; Department of Orthopedics, General Hospital of Northern Theater Command, No. 83 Wenhua Road, Shenyang 110016, Liaoning, China

**Keywords:** intervertebral disc degeneration, ZFAS1, apoptosis, ECM degradation, AAK1

## Abstract

We investigated the function of lncRNA zinc finger antisense 1 (ZFAS1) in intervertebral disc degeneration (IDD) progression *in vitro* and *in vivo*. Nucleus pulposus (NP) tissues were obtained from 20 patients with IDD. IL-1β was used to stimulate primary NP cells to establish the IDD models *in vitro*. Gene expression was determined by RT-qPCR. 5-Ethynyl-2′-deoxyuridine and flow cytometry were performed to determine cell proliferation and apoptosis, and western blotting was conducted to measure the apoptosis- and extracellular matrix (ECM)-related protein expression. Luciferase reporter assay was used to examine the interactions between the genes. We also investigated the effect of ZFAS1 in a mouse model of IDD induced by needle punctures. Our results showed that ZFAS1 expression was elevated in degenerative NP tissues and IL-1β-treated NP cells. ZFAS1 knockdown inhibited NP cell apoptosis and ECM degradation induced by IL-1β. Mechanically, ZFAS1 sponged miR-4711-5p and adaptor-associated kinase 1 (AAK1) was targeted by miR-4711-5p. Furthermore, AAK1 overexpression partially eliminated the impact of ZFAS1 depletion on NP cell proliferation, apoptosis, and ECM degradation. More importantly, the results of the *in vivo* studies confirmed the effect of silencing ZFAS1 on alleviating the symptoms of IDD mice. Overall, silencing ZFAS1 inhibits IDD progression by reducing NP cell apoptosis and ECM degradation through the miR-4711-5p/AAK1 axis.

## 1 Introduction

Lumbar intervertebral disc (IVD) degeneration (IDD) is a disabling condition that can directly cause low back pain [1]. It is a complex cascade associated with manifold factors (e.g., mechanical loading, aging, inflammation, and hormone secretion), which leads to a progressive and irreversible structural disc failure [2,3]. A series of degenerative spinal diseases, such as lumbar spinal canal stenosis, degenerative scoliosis, and lumbar disc herniation are strongly related to the pathogenesis of IDD [4]. Nucleus pulposus (NP) cell death and extracellular matrix (ECM) degradation are the main pathological changes during the progression of IDD [5]. NP cells are mainly responsible for the synthesis of ECM. Imbalance between anabolism and catabolism reduces the amount of proteoglycan and collagen [6]. However, the exact mechanisms in the pathogenesis of IDD are still uncharacterized. There are no effective therapeutic strategies to prevent degeneration of IVD [7]. Therefore, it is needed to perform basic research to elucidate the mechanisms in IDD for improvement of clinical outcomes of this disease.

As a kind of non-coding RNAs containing over 200 nucleotides, lncRNAs function as crucial regulators in various biological processes including epigenetic regulation, immune response, stem cell differentiation, and tumorigenesis [8–10]. lncRNAs are also regarded as important indicators, biomarkers, and therapeutic targets in the pathologic and physiologic processes of chondrogenesis, regeneration, and degeneration [11,12]. Evidence suggests that lncRNAs participate in the development of disease including that of IDD by acting as competitive endogenous RNAs (ceRNAs). For example, LINC00969 increases NP cell death by sponging miR-335-3p to upregulate TXNIP expression in IDD progression [13]. lncRNA HCG18 facilitates the degeneration of IVD by inhibiting NP cell growth through the miR-146a-5p/TRAF6 axis [14]. lncRNA TUG1 accelerates NP cell apoptosis and ECM degradation in IDD by sponging miR-26a to upregulate HMGB1 [15]. Zinc finger antisense 1 (ZFAS1), a lncRNA located at the chromosome 20p 13.13 locus, has been widely identified as an oncogene in tumorigenesis. ZFAS1 was found to increase inflammation and hyperplasia of fibroblast-like synoviocytes in rheumatoid arthritis [16] and aggravates spinal cord injury by facilitating cell apoptosis and inflammatory [17]. Moreover, ZFAS1 was shown to be related to increased disease severity and intensified inflammatory reaction in patients with lumbar IDD [18]. These indicate that ZFAS1 may participate in the pathogenesis of IDD.

In this study, we investigated the role of ZFAS1 in the apoptosis and ECM degradation of NP cells under IL-1β stimulation. We also investigated the effect of ZFAS1 in a mouse model of IDD. Additionally, the ceRNA network addressed by ZFAS1 was investigated.

## 2 Materials and methods

### 2.1 Patient samples

NP tissues were collected from 20 patients with IDD who underwent lumbar spine surgery (*n* = 12 female and *n* = 8 men; mean age 38 ± 9 years; ranging 30–47 years) and 20 control patients with lumbar vertebrae fractures (*n* = 10 female and *n* = 10 men; mean age 35 ± 10 years; ranging 32–50 years). Patients with IDD were clinically diagnosed as IDD by preoperative magnetic resonance imaging and computed tomography. Patients in the control group had no history of low back pain and no symptoms of IDD.


**Ethics approval and consent to participate:** This study was permitted by the Ethics Committee of General Hospital of Northern Theater Command (Liaoning, China). Informed consent was assigned by all participants before the experiment.

### 2.2 Culture of degenerative NP cells

Briefly, fresh NP tissues were kept in the solution containing 0.9% sodium chloride (S3014; Sigma-Aldrich, MO, USA), and then washed twice with phosphate-buffered saline (P5368-10PAK; Sigma-Aldrich). The samples were treated with 0.25% trypsin-EDTA (40127ES60; Yeasen Biotechnology, Shanghai, China) for 30 min and with 0.2% collagenase type II (17101015; Gibco, NY, USA) containing 0.1% FBS (10270-106; Gibco) and 1% penicillin–streptomycin mix (15140122; Gibco) for 3–4 h at 37°C. After isolation, cells were filtered through a 70 μm mesh filter (352350; BD Biosciences, NJ, USA). Primary NP cells were resuspended in Dulbecco’s Modified Eagle’s Medium (DMEM)/F12 (12400-024; Gibco) containing 20% FBS and 1% penicillin–streptomycin mix and incubated at 37°C with 5% CO_2_. The third passaged NP cells were used in the subsequent experiments. To establish the models of IDD *in vitro*, 10 ng/mL IL-1β (ALX-520-001; Enzo Life Science, NY, USA) was treated with NP cells for 6, 12, 24, and 48 h.

### 2.3 Cell transfection

Full length of ZFAS1 or adaptor-associated kinase 1 (AAK1) sequence was synthesized by RiboBio (Guangzhou, China) and inserted into the pcDNA3.1 vector (V79020; Invitrogen, CA, USA) to generate pcDNA/ZFAS1 or pcDNA/AAK1. The miR-4711-5p mimics (UGCAUCAGGCCAGAAGACAUGAG), control miRNA mimics (NC mimics; AAACACUUCAAGAGGGGUCCAUA), as well as shRNA targeting ZFAS1 (sh-ZFAS1; GAATATATATATACATATA) and scrambled control (sh-NC; TATATGTATATATATATTC) were obtained from RiboBio (Guangzhou, China). NP cells were seeded into 24-well plates at 1 × 10^7^ cells/well, and then 2 µg vectors or 50 nM synthetic oligonucleotides were transfected into NP cells. Transfection was performed by using Lipofectamine 2000 (11668; Invitrogen) according to the manufacturer’s protocol [19,20].

### 2.4 RT-qPCR

Total RNA from NP tissues and cells was extracted using TRIzol (Cat. No. 16096020, Thermo Fisher Scientific, MA, USA) and RNeasy Mini Kit (74104; Qiagen, Hilden, Germany). Reverse transcription was conducted using the PrimeScript^TM^ RT reagent Kit with gDNA Eraser (RR047A; Takara, Japan). The quantitative PCR was performed with the SYBR^®^ Premix Ex TaqTMII Kit (RR820A;Takara) on an ABIPRISM^®^ 7300 (Model Prism^®^ 7300, Kunke, Shanghai, China). The levels of miRNAs (miR-4711-5p, miR-6499-3p, miR-5580-5p, miR-3924, and miR-4269) and mRNA (ZFAS1, GADD45A, GTF3A, SCN4B, PSMB6, KRT10, AAK1, ITGA9, VN1R2, CHST14, and ALG13) were calculated using the 2^−∆∆Ct^ method with U6 and GAPDH as internal controls. The sequences of primers are shown in Table 1.

**Table 1:** The sequences of primers for RT-qPCR

**Human**	**Primer sequence (5′-3′)**
**Primer sequences used for RT-qPCR**	
ZFAS1	F: GAAGAGGGAGTCACCACTG
ZFAS1	R: CCAACAATAAACTCGTCAGGAG
miR-4711-5p	F: GCGCTGCATCAGGCCAGAAGATT
miR-4711-5p	R: GTGCAGGGTCCGAGGT
miR-6499-3p	F: GCGCAGCAGTGTTTGTTTTGTT
miR-6499-3p	R: GTGCAGGGTCCGAGGT
miR-5580-5p	F: GCGCTGCTGGCTCATTTCATTT
miR-5580-5p	R: GTGCAGGGTCCGAGGT
miR-3924	F: GCGCATATGTATATGTGACTTT
miR-3924	R: GTGCAGGGTCCGAGGT
miR-4269	F: GCAGGCACAGACAGCCCTG
miR-4269	R: GAACATGTCTGCGTATCTC
GADD45A	F: ACCGAAAGGATGGATAAGGT
GADD45A	R: CACAACACCACGTTATCGG
GTF3A	F: CAATGAACCTCTATTCAAGTGTACC
GTF3A	R: CCTTGGCATGTCGTTTCAG
SCN4B	F: CGCATTCAAGATTCTCATAGAG
SCN4B	R: AGTAGAGCCTACCAGAGTG
PSMB6	F: GAAGTTTCCACTGGGACCA
PSMB6	R: AGTGGTTGTTCTGGAGTCC
KRT10	F: ACTGATAATGCCAACATCCT
KRT10	R: CTCATTCTCATACTTCAGCCT
AAK1	F: TAAGTCCAAGTCTGCAACCA
AAK1	R: GACCCTTCTGAAGGATTATAAACG
ITGA9	F: TGATGGGTTCCCAGATGTG
ITGA9	R: GCATCACCATGATAGATATAGACC
VN1R2	F: GGGCTGGACCCTACACTATTC
VN1R2	R: GGATCTTGGCTTGTATCCCCT
CHST14	F: GTGCTGCCTAAGTATATCCTG
CHST14	R: TGGTGACATTAGGCAGTGG
ALG13	F: TATTAGTCACGCAGGTGCA
ALG13	R: TTTGTGTAGCTGCTTTGCC
GAPDH	F: TCAAGATCATCAGCAATGCC
GAPDH	R: CGATACCAAAGTTGTCATGGA
U6	F: ATACAGAGAAAGTTAGCACGG
U6	R: GGAATGCTTCAAAGAGTTGTG
**Mouse**	
ZFAS1	F: AGCGTTTGCTTTGTTCCC
ZFAS1	R: CTCCCTCGATGCCCTTCT
AAK1	F: GGGAGATCCAGATCATGAGAG
AAK1	R: TGAGAACTTCCCAGACGTC
GAPDH	F: ACTCTTCCACCTTCGATGC
GAPDH	R: CCGTATTCATTGTCATACCAGG
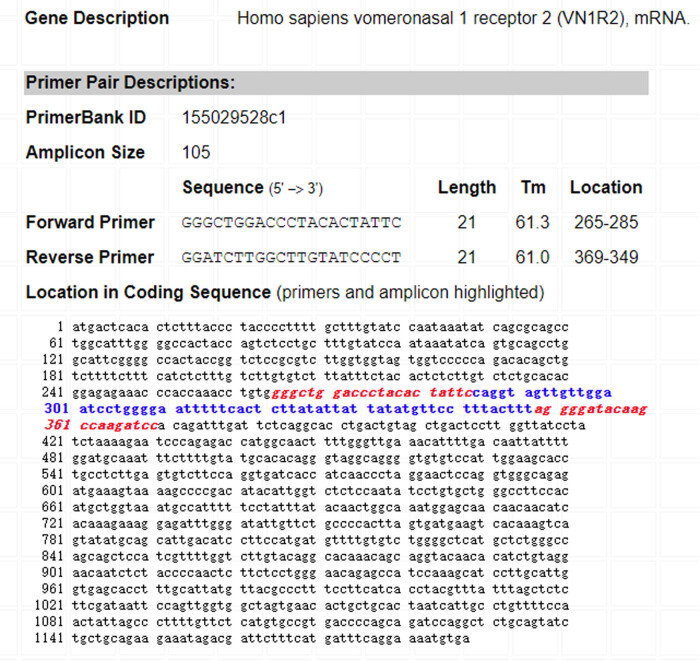	

### 2.5 Western blotting

NP tissues or cells were lysed using RIPA buffer (R0278; Sigma-Aldrich). Total protein was separated by 12% sodium dodecyl sulphate-polyacrylamide (SDS-PAGE; P0012A; Beyotime, Shanghai) electrophoresis and then was transferred to polyvinylidene difluoride membranes (IPVH00010; Millipore, Billerica, MA, USA). Western blotting was performed as previously recorded [21] with primary antibodies: Bax (ab32503; 1:1,000, Abcam), Bcl-2 (ab182858; 1:2,000), cleaved caspase-3 (9661; 1:1,000, Cell Signal Technology), cleaved caspase-9 (9508; 1:1,000), AAK1 (61527; 1:1,000), Collagen II (ab34712; 1:1,000), Aggrecan (MA5-42646; 1:1,000, Invitrogen), MMP-3 (ab52915; 1:1,000), MMP-13 (a39012; 1:3,000), and GAPDH (ab181602; 1:10,000). After the first step incubation, the samples were further incubated with secondary antibodies for 1 h at room temperature. The membranes were washed and observed using ECL Western Blotting Substrate (32106; Thermo Fisher Scientific). Signal quantification was performed using ImageJ software (version 1.8.0; National Institutes of Health).

### 2.6 Fluorescent *in situ* hybridization (FISH)

The localization of ZFAS1 in NP cells was detected by performing FISH as previously described [22]. ZFAS1 probes were designed and synthesized by RiboBio (Guangzhou, China). The signals of the probes were detected by FISH kit (C10910; RiboBio) according to the manufacturer’s instructions. The images were acquired with a Nikon A1Si laser scanning confocal microscope (Nikon Instruments Inc., Japan).

### 2.7 5-Ethynyl-2′-deoxyuridine (EdU) assay

Briefly, NP cells were seeded into 24-well plates at 5 × 10^4^ cells/well. Next, 10 µmol/L EdU reagent was added to each well and incubated for 2 h at room temperature at 37°C according to the protocol of EdU fluorescence staining cell proliferation kit (KGA331-1000; Nanjing KeyGen Biotech, Co., Ltd). Cells were washed and fixed with 4% paraformaldehyde (P6148; Sigma-Aldrich) for 30 min at room temperature. After the staining solution was washed, 10 µmol/L DAPI (F6057; Sigma-Aldrich) was used to stain the nucleus for 5 min at room temperature. Fluorescence images were obtained using an IX73 fluorescence microscope (Olympus Corporation, Japan) and EdU-positive cells were counted using ImageJ software (version 1.8.0; National Institutes of Health).

### 2.8 Flow cytometry

The apoptosis of NP cells was examined using Annexin V-FITC/PI apoptosis detection kit (A211-01; Novizan Biotechnology Co., Nanjing, China). NP cells in 6-well plates at 1 × 10^5^ cells/well were cultured overnight at 37°C. Cells were cultured at 80% confluence and suspended in binding buffer at 1 × 10^6^ cells/mL. Next, 10 μL Annexin V-FITC and 10 μL PI were added into cells and maintained in the dark for 25 min. Finally, the apoptosis was detected using Elite ESP flow cytometry (FACSCalibur, Becton‑Dickinson Immunocytometry Systems, CA, USA) and data were analyzed using Cell Quest Pro software (version 5.1; BD Biosciences).

### 2.9 Luciferase reporter assay

The binding sequence of miR-4711-5p at the ZFAS1 or AAK1 3′-UTR was predicted in DIANA database or TargetScan database. The cDNA sequence of wild type ZFAS1 or AAK1 3′-UTR was inserted into the pmirGLO vector (E1330; Promega, USA), and a luciferase reporter plasmid ZFAS1-Wt/AAK1-Wt was established. The mutated ZFAS1 or AAK1 3′-UTR sequence was also cloned into the same vector to generate ZFAS1-Mut/AAK1-Mut luciferase reporter plasmids. HEK293T cells (ATCC, USA) were cultured in 48-well plates at 2 × 10^3^ cells/well. Next, 2.5 µg luciferase reporter plasmids were transfected into HEK293T cells together with 50 nM miR-4711-5p mimics or NC mimics. After 48 h, the firefly luciferase activity was examined using Dual-Luciferase Reporter Assay System (E1960; Promega).

### 2.10 Construction of ZFAS1 knockdown vector

Adeno-associated virus9 (AAV9) vector was used to carry a shRNA fragment to silence ZFAS1 (shZFAS1). The sh-NC and sh-ZFAS1 were constructed by GenePharma (Shanghai, China). Virus solution (1.3 × 10^10^ puf/mL) was used to treat mice.

### 2.11 Mouse model of IDD

Forty C57BL/6J mice (aged 10–14 weeks; Vital River Co. Ltd, Beijing, China) were used for animal experiments. All animals were housed in a specific pathogen free environment with 12 h light or dark cycle (humidity: 50–60%; temperature: 22°C), and they had free access to water and food. Ten mice in the sham group were free of surgical intervention, and the remaining 30 mice received surgery. Animals were intraperitoneally injected with dimethylrazine (10 mg/kg) and ketamine (90 mg/kg) for anesthesia. A sagittal small skin incision was performed from Co6 to Co8 to help locate the disc position for needle insertion in the tail. Subsequently, Co6–Co7 coccygeal discs were punctured using a needle. The syringe needle was inserted into the Co6–Co7 disc along the vertical direction and then rotated in the axial direction by 180° and then was held for 10 s. The puncture was made parallel to the endplates through the annulus fibrosus into the NP using a 31-G needle (Hamilton, Switzerland), which was inserted 1.5 mm into the disc to depressurize the nucleus. The other segments were left undisturbed for contrast. The wound was closed.

After 1 week of IVD puncture, animals receiving surgery were randomly divided into three groups: IDD, IDD-AAV-sh-NC, and IDD-AAV-sh-ZFAS1 (ten mice for each group). After anesthesia, the previously punctured disc from the left side was exposed with a small incision. Next, 3 μL AAV-sh-NC or AAV-sh-ZFAS1 (AAV: 1.3 × 10^10^ pfu/mL) was inserted within the disk capsule intradiscally using a 33-gauge needle tip (Hamilton), which was connected to a microsyringe (Hamilton). Four weeks after injection, animals were euthanized, and the discs were harvested for histological analysis of hematoxylin–eosin. Animal experiments were strictly in accordance with the Guide to the Management and Use of Laboratory Animals issued by the National Institutes of Health.


**Ethics approval:** The animal study was reviewed and approved by the Animal Ethics Committee of General Hospital of Northern Theater Command (Shenyang, China; ethics approval number: Y (2020) 010).

### 2.12 Histological examination

The discs from mice were fixed with 4% paraformaldehyde (P6148; Sigma-Aldrich) for 1 day, demineralized in 10% ethylenediaminetetraacetic acid (Wako Osaka, Japan) for 7 days, embedded in paraffin, and cut to obtain a mid-sagittal 7 µm section for histomorphology. Midsagittal sections were stained with hematoxylin (30002; Muto Tokyo, Japan). The histological images were analyzed using an Olympus BX51 microscope. A modified grading system was used to assess the degree of degeneration, based on the grading system of the rabbits and human beings [23,24]. The grading scores ranged from 1 to 4 for both annulus fibrosus and NP. The scores of both annulus fibrosus and NP were summed to evaluate the degree of degeneration.

### 2.13 Statistical analysis

Data are expressed as mean ± standard deviation, except for histological scores, which were reported as the median with the range. Statistical analysis was performed using SPSS 18.0 software (IBM Corp., Armonk, NY, Chicago, USA). All experiments were performed three times, independently. Student’s *t*-test or one-way ANOVA with the Tukey–Kramer *post-hoc* test was performed with a significance level of *p* < 0.050 with the normality assumption.

## 3 Results

### 3.1 Effects of silencing ZFAS1 on NP cell apoptosis and ECM degradation

**Figure 1:**
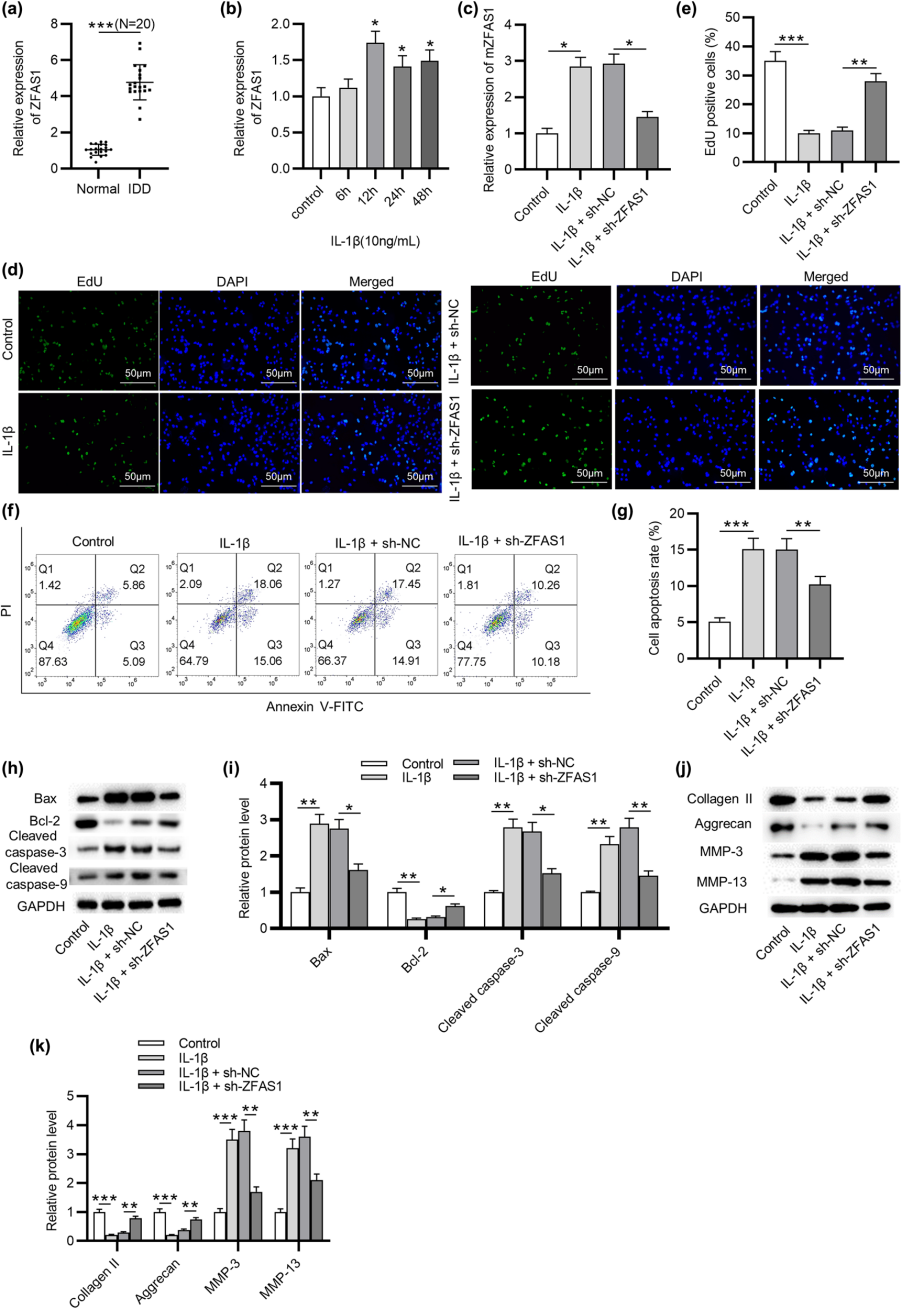
Effect of silencing ZFAS1 on NP cell apoptosis and ECM degradation. (a) RT-qPCR analysis of ZFAS1 expression in NP tissues of IDD patients. (b) ZFAS1 expression in NP cells treated with 10 ng/mL IL-1β for 6, 12, 24, and 48 h. NP cells were then treated with 10 ng/mL IL-1β for 12 h. (c) ZFAS1 expression in IL-1β-treated NP cells transfected with sh-NC or sh-ZFAS1. (d and e) EdU assay of cell proliferation in IL-1β-treated NP cells transfected with sh-NC or sh-ZFAS1. (f and g) Flow cytometry of cell apoptosis in IL-1β-treated NP cells transfected with sh-NC or sh-ZFAS1. (h–k) Western blotting analysis of the expression of Bax, cleaved caspase-3, cleaved caspae-9, Bcl-2, Collagen II, Aggrecan, MMP-3, and MMP-13 in IL-1β-treated NP cells transfected with sh-NC or sh-ZFAS1. ^*^ *p* < 0.05, ^**^ *p* < 0.01, ^***^ *p* < 0.001.

To elucidate the function of ZFAS1 in the IDD pathogenesis, we measured the ZFAS1 level in NP tissues from patients with lumbar vertebrae fractures (normal control) or patients with IDD using RT-qPCR. The results showed that NP tissues in the IDD group had significantly upregulated ZFAS1 expression compared to those in the normal group (Figure 1a). Many reports have indicated the function of IL-1β in inducing the loss and degeneration of NP cells [25,26]. In this study, 10 ng/mL IL-1β was used to stimulate NP cells for 6, 12, 24, or 48 h. As shown in Figure 1b, the ZFAS1 level peaked at 12 h after IL-1β stimulation. Therefore, the subsequent experiments were performed in NP cells treated with 10 ng/mL IL-1β for 12 h. Interference of ZFAS1 expression in NP cells was performed to explore whether ZFAS1 is involved in IL-1β-mediated NP cell survival, apoptosis, and ECM degradation. The RT-qPCR results indicated that ZFAS1 expression was significantly reduced in sh-ZFAS1-transfected NP cells compared to sh-NC-transfected cells (Figure 1c). EdU assay in Figure 1d and e exhibited that NP cell proliferation treated with IL-1β was significantly inhibited compared to the control group. However, transfection with sh-ZFAS1 restored the proliferation of NP cells inhibited by IL-1β. Additionally, IL-1β markedly accelerated the apoptosis of NP cells, while the apoptosis was suppressed after ZFAS1 was knocked down (Figure 1f and g). The impact of IL-1β on proapoptotic protein (Bax, cleaved caspase-3, cleaved caspase-9) and antiapoptotic protein Bcl-2 was also reversed by transfection with sh-ZFAS1 (Figure 1h and i). Furthermore, we evaluated the function of ZFAS1 on ECM degradation in NP cells. The western blotting results demonstrated that, after IL-1β treatment, collagen II and aggrecan protein levels were downregulated, and MMP-3 and MMP-13 protein levels were elevated, while these changes mediated by IL-1β were reversed by sh-ZFAS1 (Figure 1j and k). These results suggested that ZFAS1 knockdown mitigated IL-1β-induced apoptosis and ECM degradation in NP cells.

### 3.2 ZFSA1 interacts with miR-4711-5p

**Figure 2:**
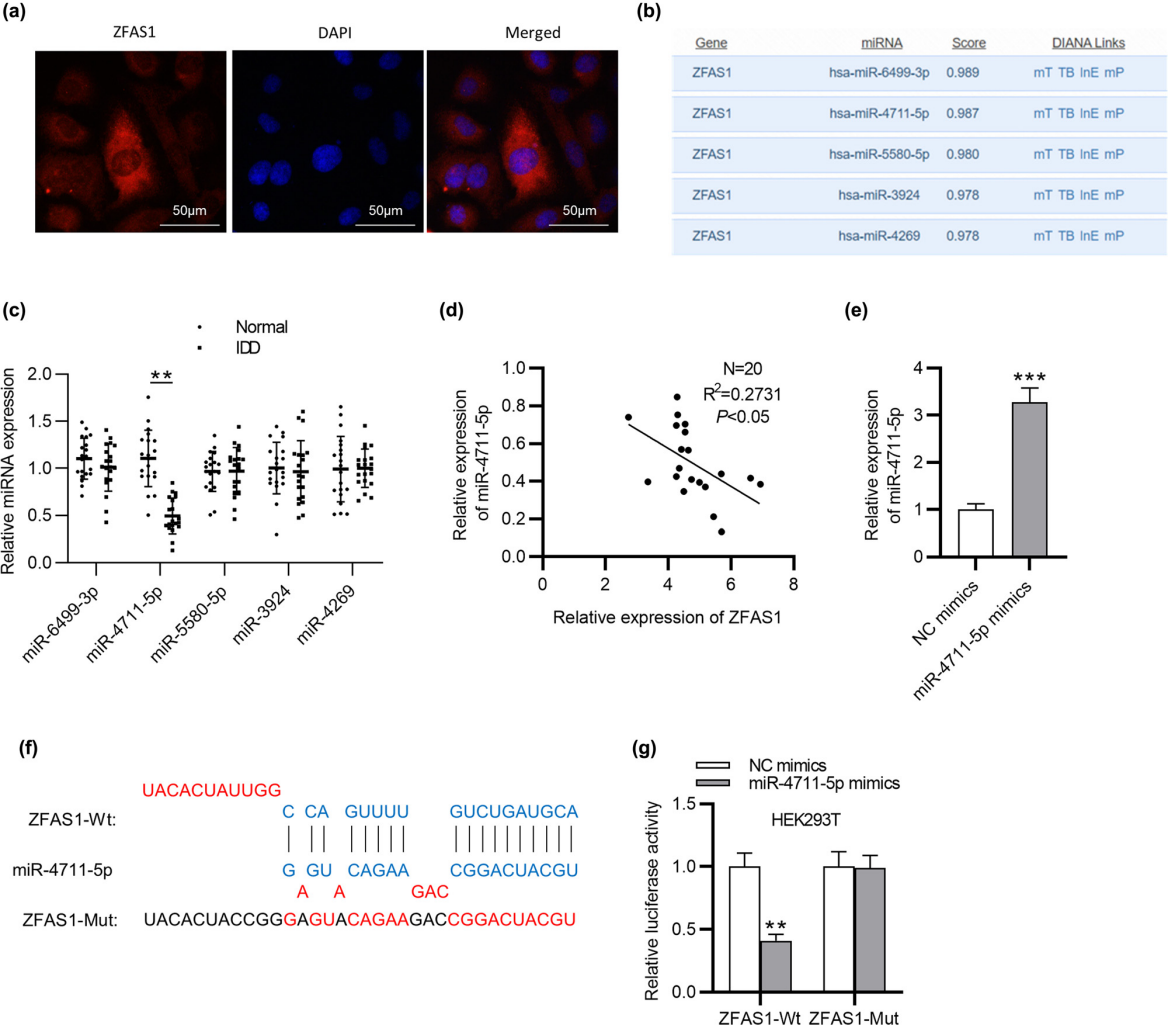
ZFSA1 interacts with miR-4711-5p. (a) FISH assay of the localization of ZFAS1 in NP cells. (b) The miRNAs that have complementary base pairing with ZFAS1 in DIANA database. (c) RT-qPCR analysis of miRNA expression in NP tissues of IDD patients. (d) Correlation between ZFAS1 and miR-4711-5p expression in NP tissues of IDD patients. (e) RT-qPCR analysis of miR-4711-5p mimics transfection efficiency in NP cells. (f) Binding sequence of miR-4711-5p complementary to ZFAS1. (g) Luciferase activity in HEK293T cells transfected with ZFAS1-Wt or ZFAS1-Mut reporter vector together with miR-4711-5p mimics. ^**^ *p* < 0.01, ^***^ *p* < 0.001.

To explore the mechanisms of ZFAS1 in IDD, we performed FISH to probe the localization of ZFAS1 in NP cells. As presented in Figure 2a, ZFAS1 expression showed a significant distribution in the cytoplasm. Subsequently, the DIANA online tool was used to identify the MicroRNAs (miRNAs) that have complementary base pairing with ZFAS1. Based on the binding score, five miRNAs (miR-6499-3p, miR-4711-5p, miR-5580-5p, miR-3924, and miR-4269) are shown (Figure 2b). Next, we assessed the expression of these candidate mRNAs in NP tissues of IDD patients. The RT-qPCR results indicated that miR-4711-5p exhibited a significant downregulation in IDD, while the other miRNAs had no significant change in the expression levels (Figure 2c). Moreover, the miR-4711-5p level was negatively associated with the ZFAS1 level in IDD samples (Figure 2d). RT-qPCR revealed that miR-4711-5p expression was markedly increased in NP cells after miR-4711-5p mimics were transfected (Figure 2e). The DIANA database shows that miR-4711-5p contains a binding sequence complementary to ZFAS1 (Figure 2f). Luciferase reporter assay indicated that miR-4711-5p overexpression inhibited the luciferase activity of ZFAS1-Wt plasmids without affecting that of ZFAS1-Mut plasmids (Figure 2g). Therefore, ZFAS1 could bind to miR-4711-5p.

### 3.3 AAK1 is targeted by miR-4711-5p

**Figure 3:**
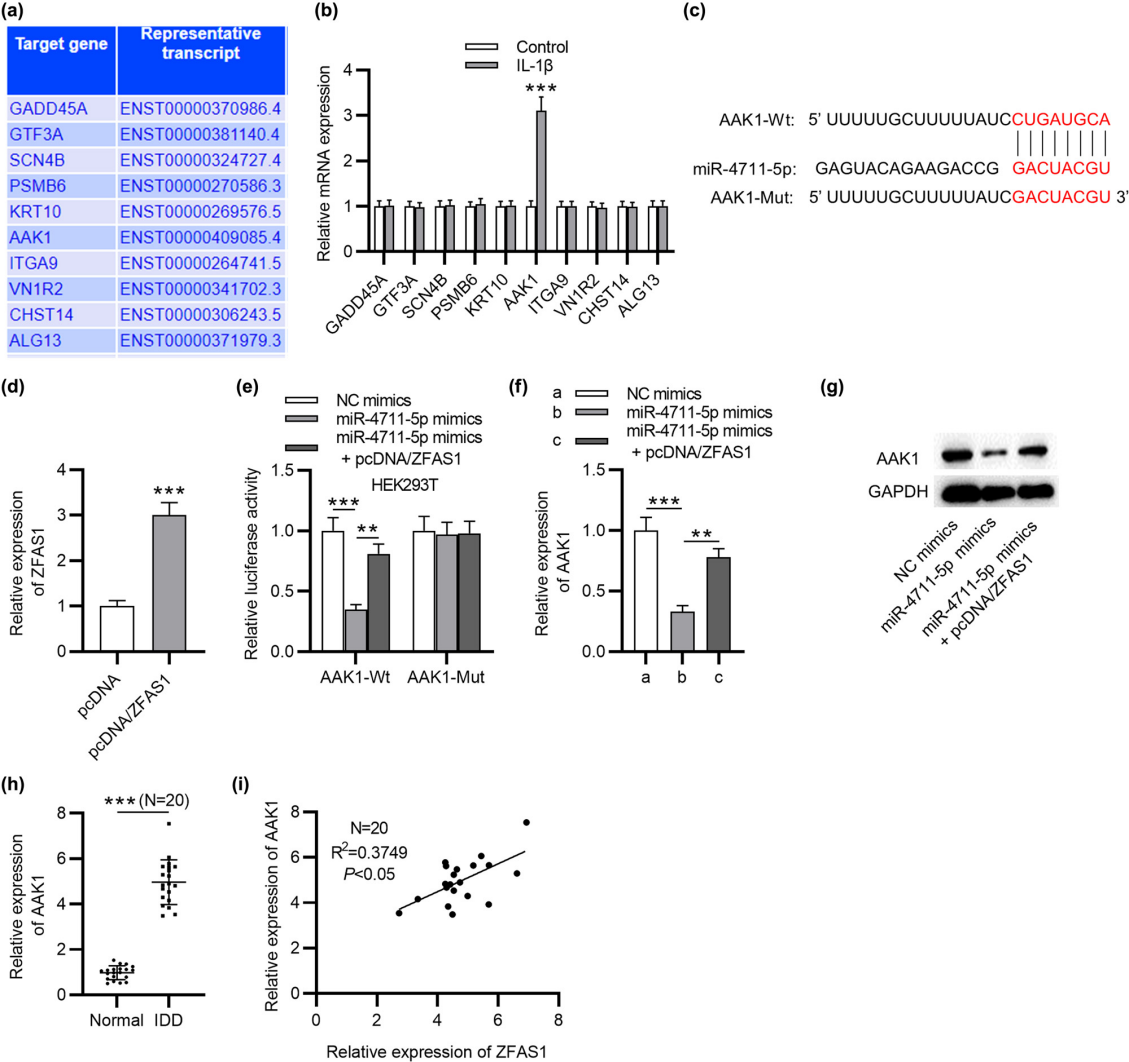
AAK1 is targeted by miR-4711-5p. (a) Ten putative mRNAs capable of binding to miR-4711-5p were found in TargetScan database. (b) The mRNA expression in NP cells treated with 10 ng/mL IL-1β. (c) Binding sequence of miR-4711-5p complementary to AAK1. (d) Overexpression efficiency of pcDNA/ZFAS1 in NP cells. (e) Luciferase activity in HEK293T cells transfected with AAK1-Wt or AAK1-Mut reporter vector together with the indicated plasmids. (f and g) RT-qPCR and western blotting analysis of AAK1 expression in NP cells transfected with NC mimics, miR-4711-5p mimics, and miR-4711-5p mimics + pcDNA/ZFAS1. (h) RT-qPCR analysis of AAK1 expression in NP tissues of IDD patients. (i) Correlation between AAK1 and miR-4711-5p expression in NP tissues of IDD patients. ^**^ *p* < 0.01, ^***^ *p* < 0.001.

Next, the TargeScan database was examined to identify the downstream targets of miR-4711-5p. As exhibited in Figure 3a, ten mRNAs possibly capable of binding to miR-4711-5p were found based on the cumulative weighted context++ score. The RT-qPCR results revealed that AAK1 had the most significant upregulation in IL-1β-treated NP cells among all mRNA candidates (Figure 3b). The binding sequence of miR-4711-5p complementary to AAK1 is presented in Figure 3c. The overexpression efficiency of pcDNA/ZFAS1 was tested and confirmed by RT-qPCR (Figure 3d). Luciferase reporter experiment showed that miR-4711-5p overexpression suppressed the luciferase activity of the vectors containing AAK1-Wt in HEK293T cells, and this effect was attenuated after ZFAS1 was upregulated (Figure 3e). In parallel, ZFAS1 overexpression restored the AAK1 mRNA and protein expression decreased by miR-4711-5p overexpression (Figure 3f and g). We further discovered that AAK1 expression was high in NP tissues of IDD patients, which was positively associated with ZFAS1 expression (Figure 3h and i). Overall, ZFAS1 upregulated AAK1 through controlling the availability of miR-4711-5p.

### 3.4 ZFAS1 facilitates NP cell apoptosis and ECM degradation by AAK1

**Figure 4:**
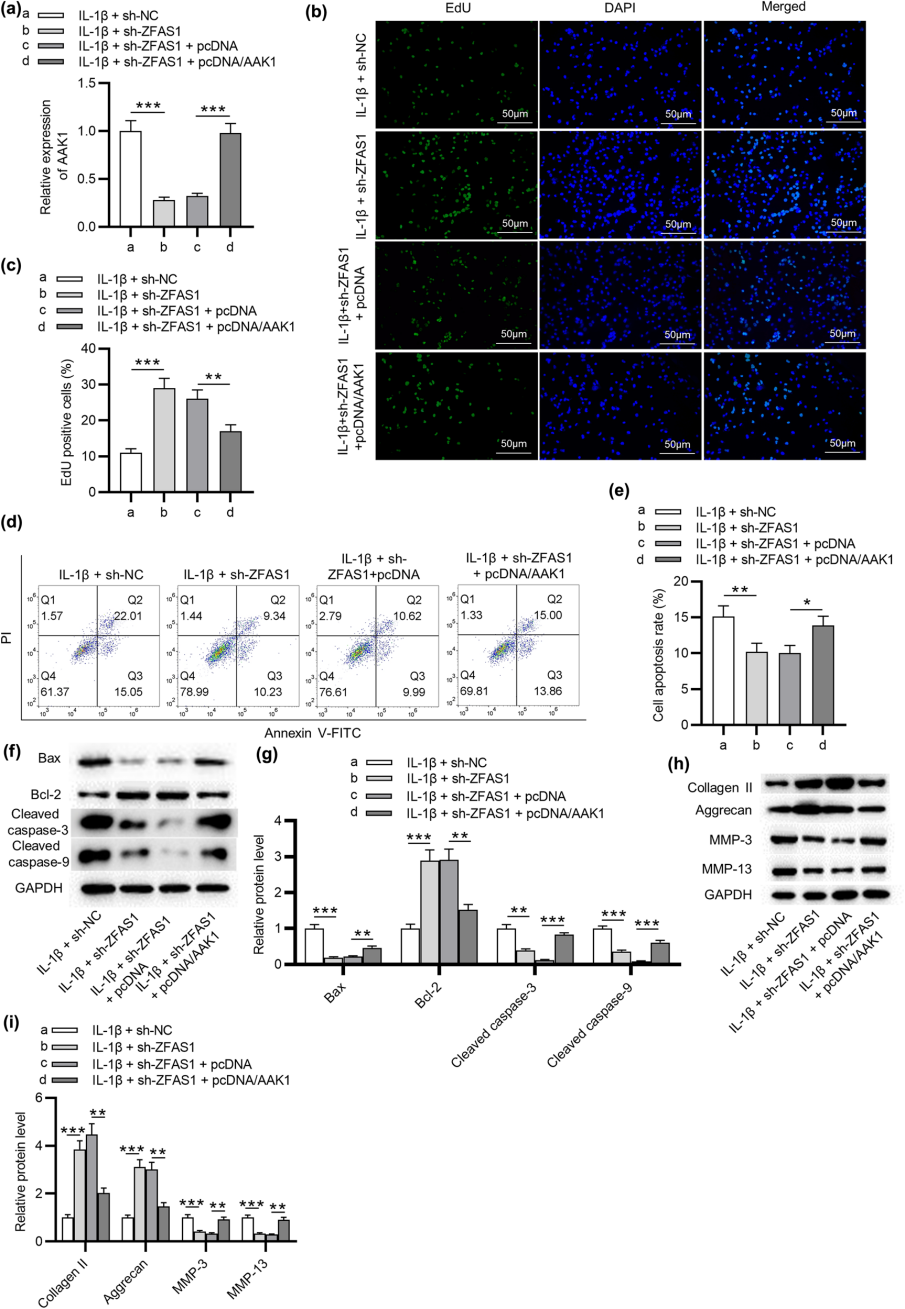
ZFAS1 facilitates NP cell apoptosis and ECM degradation by AAK1. NP cells were then treated with IL-1β + sh-NC, IL-1β + sh-ZFAS1, IL-1β + pcDNA, and IL-1β + pcDNA/AAK1M, respectively. (a) AAK1 expression in NP cells. (b and c) EdU assay of cell proliferation in each group. (d and e) Flow cytometry analysis of cell apoptosis in each group. (f–i) Western blotting analysis of the expression of Bax, Bcl-2, Collagen II, Aggrecan, MMP-3, and MMP-13 in each group. ^*^ *p* < 0.05, ^**^ *p* < 0.01, ^***^ *p* < 0.001.

Rescue experiments were conducted in IL-1β-treated NP cells transfected with sh-NC, sh-ZFAS1, sh-ZFAS1 + pcDNA, and sh-ZFAS1 + pcDNA/AAK1. As illustrated in Figure 4a, the AAK1 level was decreased in sh-ZFAS1-transfected cells and was restored after pcDNA/AAK1 transfection. EdU assay indicated that AAK1 overexpression abrogated the promotive effect of silencing ZFAS1 on NP cell proliferation (Figure 4b and c). Analyses of flow cytometry and apoptosis-related protein levels showed that the apoptosis of NP cells inhibited by ZFAS1 depletion was restored by overexpression of AAK1 (Figure 4d and g). Moreover, the effect of ZFAS1 knockdown on the Collagen II, Aggrecan, MMP-3, and MMP-13 protein expression was eliminated by overexpression of AAK1 (Figure 4h and i). These findings demonstrated that ZFAS1 accelerates NP cell apoptosis and ECM degradation by elevating AAK1.

### 3.5 ZFAS1 knockdown alleviates IDD *in vivo*

**Figure 5:**
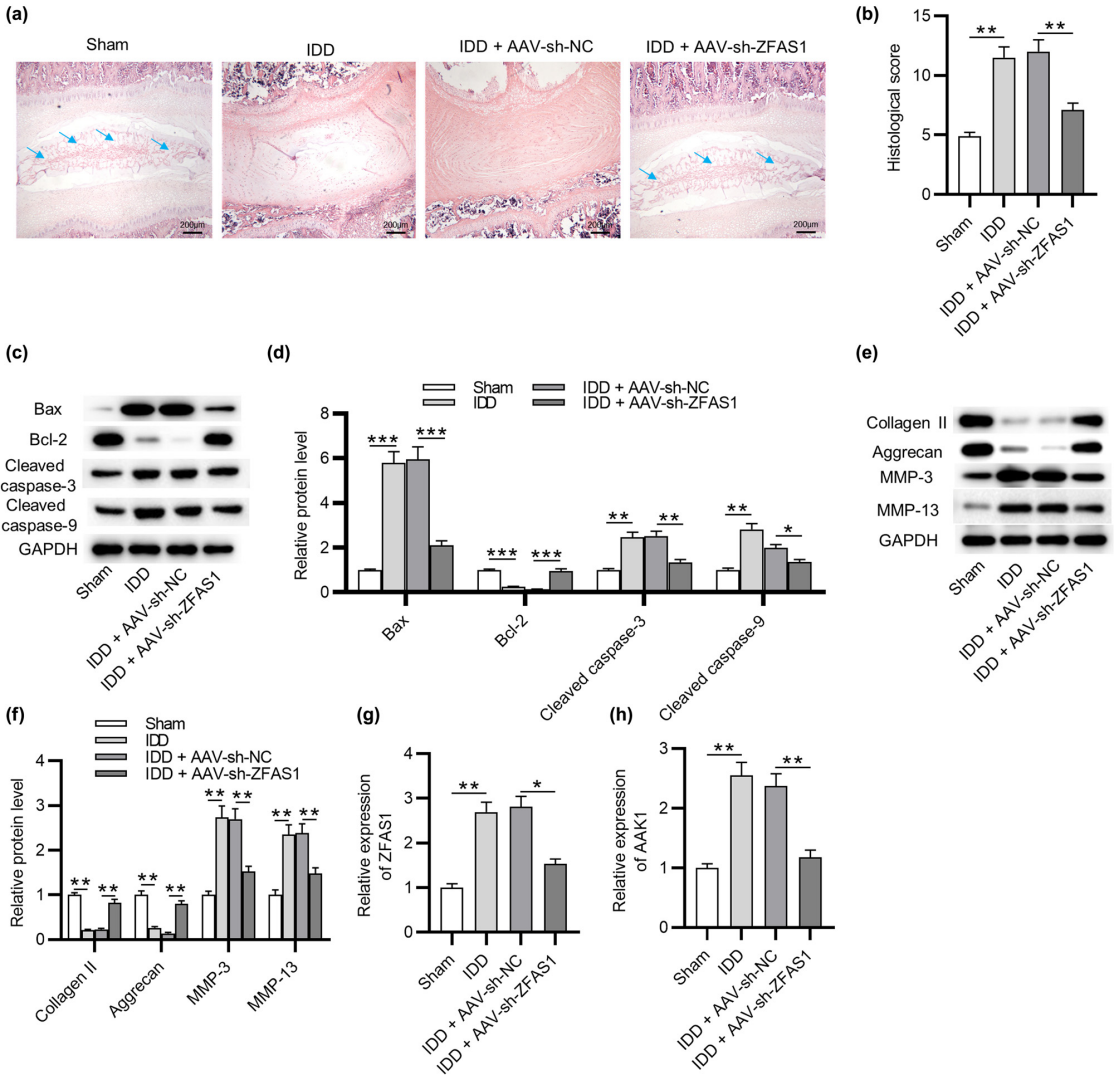
ZFAS1 knockdown alleviates IDD *in vivo*. (a) Hematoxylin–eosin staining of intervertebral disc tissues of mice. *N* = 10. (b) Histological score in each group. *N* = 10. (c–f) Western blotting analysis of the expression of Bax, Bcl-2, Collagen II, Aggrecan, MMP-3, and MMP-13 in intervertebral disc tissues of mice. *N* = 10. (g and h) RT-qPCR analysis of the expression of ZFAS1 and AAK1 in intervertebral disc tissues of mice. *N* = 10. ^*^ *p* < 0.05, ^**^ *p* < 0.01, ^***^ *p* < 0.001.

Hematoxylin–eosin staining of intervertebral disc tissues showed that the IDD mouse model was successfully established. This was shown by increased collagen fibers and disappearance of the reticular structure in the NP in IDD mice. After injection of AAV-sh-NC, IDD mice showed symptoms such as loss of chondrocytes, smaller nuclear size with deepened staining, and blurred or even absent cell cytoplasm structure. However, these symptoms were alleviated in IDD mice injected with AAV-sh-ZFAS1 (Figure 5a). A scoring system was used to assess the grading of IDD, showing that AAV-sh-ZFAS1 significantly reduced histological score of IDD mice (Figure 5b). Moreover, the protein levels of Bax, cleaved caspase-3, cleaved caspase-9, MMP-3, and MMP-13 were increased in DDI mice, and the protein levels of Bcl-2, collagen II, and aggrecan were decreased in DDI mice, while these changes were reversed by AAV-sh-ZFAS1 (Figure 5c–f). Additionally, ZFAS1 and AAK1 expression was upregulated in DDI mice, while AAV-sh-ZFAS1 reversed these changes (Figure 5g and h).

## 4 Discussion

Recently, accumulating reports have shown the dysregulation of lncRNAs in IDD progression [27]. Chen et al. found that multiple lncRNAs such as AC005082.12, RP11-363G2.4, and LINC00917 were differentially expressed in degenerative NP tissues and might play key roles in the development of IDD [28]. Microarray data profiling investigated by Wan et al. showed 67 up and 49 down lncRNAs in human NP tissues, and lncRNA RP11-296A18.3 possibly upregulates Fas-associated protein factor 1 expression to accelerate the death of disc degenerative NP cells [29]. lncRNA HOTAIR [30], TUG1 [31], and PART1 [32] were reported to regulate NP cell senescence, ECM degradation, and apoptosis in IDD progression. Thus, investigating the mechanisms of how lncRNAs mediate IDD progression may help discover novel therapeutic targets for this disease. Many reports have revealed the function of IL-1β in inducing the loss and degeneration of NP cells [25,26]. Here, IL-1β was utilized to stimulate primary NP cells *in vitro*. The results showed a significant reduction in cell proliferation and increase in cell apoptosis and ECM degradation. Additionally, IL-1β stimulation upregulated the ZFAS1 expression in NP cells. A previous report indicated that lncRNA ZFAS1 was overexpressed in IDD patients compared to control, and it was related to increased disease severity and intensified inflammatory reaction in patients with lumbar IDD [18]. The present study also confirmed the upregulation of ZFAS1 in NP tissues from IDD patients, implying the possible involvement of ZFAS1 in IDD. We found that interference of ZFAS1 expression restored cell proliferation and prevented the apoptosis and ECM degradation in IL-1β-treated NP cells. More importantly, the results of the *in vivo* studies confirmed the effect of silencing ZFAS1 on alleviating the symptoms of IDD mice.

miRNAs can control protein expression by targeting the mRNA 3′-UTR, playing key roles in physiological and pathological processes [33]. Abnormal miRNA expression is found in types of musculoskeletal diseases, such as rheumatoid arthritis and osteoporosis [34,35]. Evidence suggests that miRNAs are implicated in the process of inducing IDD [36,37]. lncRNA can play a ceRNA role to control downstream gene expression by sponging miRNAs in pathophysiological processes [38]. As an important “sponge,” ZFAS1 competitively interacts with multiple miRNAs. As previously reported, ZFAS1 depletion mitigates rheumatoid arthritis-like symptoms via miR-296-5p-dependent suppression of MMP-15 [39]. ZFAS1 regulates cell phenotypes and inflammatory reaction in fibroblast-like synoviocytes by targeting the miR-2682-5p/ADAMTS9 axis [16]. The present study discovered a predicted miR-4711-5p binding site at the ZFAS1 sequence using online predication database. Luciferase reporter experiments further corroborated the targeted relationship between miR-4711-5p and ZFAS1. Our results indicated that miR-4711-5p exhibited a downregulated expression in NP tissues from IDD patients. We concluded that ZFAS1 may exert ceRNA function in IDD by binding to miR-4711-5p. However, the function of miR-4711-5p in IDD needs to be further elucidated in the future.

Furthermore, we confirmed that miR-4711-5p directly targeted AAK1 mRNA and suppressed the AAK1 expression. AAK1 belongs to the Ark/Prk family and is a key regulatory protein in controlling clathrin-coated endocytosis [40]. A study showed that dysfunction of AAK1 led dysregulation of synaptic vesicle recycling, which was related to the pathology of amyotrophic lateral sclerosis [41]. Inhibition of AAK1 prevents the spontaneous action potentials in the spinal cord of rats with chronic constriction injury, which is considered a new strategy to treat neuropathic pain [42]. Additionally, a locus on chromosome 2 spanning AAK1 is associated with the occurrence of lumbar spinal stenosis [43]. Currently, we found that AAK1 was overexpressed in IL-1β-treated NP cells and IDD patients. AAK1 expression was positively associated with ZFAS1 expression and negatively with miR-4711-5p expression. This demonstrated that ZFAS1 upregulated AAK1 by controlling the availability of miR-4711-5p. Additionally, AAK1 overexpression blocked the inhibitory effect of silencing ZFAS1 on NP cell apoptosis and ECM degradation, and AAK1 was upregulated in DDI mice, suggesting that AAK1 may promote IDD progression.

Collectively, our study is the first to show the functional epigenic regulation of the ZFAS1/miR-4711-5p/AAK1 axis in cell apoptosis and ECM degradation under the IDD condition, which may provide new understanding of the pathogenesis of degenerative disease. Moreover, the *in vivo* effects of ZFAS1 in DDI mice further persuade our findings.
